# Multiple Basal Cell Carcinomas in an Adolescent with an MLPA-Detected *PTCH1*-Region Deletion and a Gorlin-Goltz Phenotype

**DOI:** 10.2478/bjmg-2026-0013

**Published:** 2026-10-14

**Authors:** J Mitrova Telenta, K Risteska, I Dohcheva Karajovanov, H Breshkovska, S Duma, E Mircevska Arsovska, M Dimova, V Simeonovski, D Plaseska-Karanfilskа, B Ahtarova

**Affiliations:** University Clinic of Dermatology, Faculty of Medicine, “Ss. Cyril and Methodius” University, Skopje, R. N. Macedonia; PHI General Hospital Ohrid, Ohrid, R.N. Macedonia; Research Center for Genetics Engineering and Biotechnology” Georgi Efremov”, Macedonian Academy of Sciences and Arts, Skopje, R.N. Macedonia; Primaderma-private practice, Strumica, R.N. Macedonia

**Keywords:** Gorlin-Goltz syndrome, nevoid basal cell carcinoma syndrome, basal cell carcinoma, *PTCH1*, 9q22.3 deletion, dermoscopy, Hedgehog signaling, genodermatosis, MLPA

## Abstract

Gorlin-Goltz syndrome, also known as nevoid basal cell carcinoma syndrome or basal cell naevus syndrome, is a rare hereditary cancer-predisposition disorder most often caused by disruption of the Hedgehog signaling pathway, particularly alterations of *PTCH1*. Classical disease is characterized by multiple or early-onset basal cell carcinomas, odontogenic keratocysts, palmoplantar pits, skeletal anomalies and intracranial calcifications. Deletions involving chromosome region 9q22.3 may create an overlapping but broader phenotype because the deleted interval may include *PTCH1* together with neighboring genes.

Hereby we report a 15-year-old female patient with developmental disability, including a specific speech articulation disorder, who presented with multiple facial erosions, scars, milia, papulo-nodular lesions and palmar pitting. Dermoscopy demonstrated arborizing vessels, blue-gray ovoid nests, brown dots, ulceration, white scales and shiny white perpendicular lines. Surgical excision of selected lesions was performed, and histopathology confirmed basal cell carcinoma in three specimens, reported as carcinoma basocellulare. Additional findings included a nasal-root dermoid cyst excised in early childhood, strabismus, conductive hearing loss, kyphoscoliosis and a kinked aorta. Multiplex ligation-dependent probe amplification (MLPA) using the SALSA MLPA Probemix P245-A2 Microdeletion Syndromes-1 (MRC-Holland, Amsterdam, The Netherlands) demonstrated a deletion involving the *PTCH1* region at chromosome 9q22.3.

This case is highly suggestive of a *PTCH1*-region deletion presenting with a Gorlin-Goltz phenotype, with additional extracutaneous findings that may reflect involvement of the wider 9q22.3 region; because MLPA cannot delineate deletion boundaries or confirm loss of neighboring genes, this broader interpretation remains presumptive pending array-based confirmation. The occurrence of multiple basal cell carcinomas in adolescence should prompt evaluation for inherited tumor-predisposition syndromes. When extracutaneous developmental or congenital abnormalities are present, genetic testing capable of detecting and mapping copy-number variants should be considered.

## INTRODUCTION

Gorlin-Goltz syndrome (GGS), also termed nevoid basal cell carcinoma syndrome (NBCCS) or basal cell naevus syndrome (BCNS; OMIM #109400) is a genetic disorder linked to *PTCH1*, a tumor suppressor gene, located on chromosome 9q22.3. It represents a rare multisystemic disorder characterized by developmental anomalies and predisposition to multiple neoplasms. It was first delineated by Gorlin and Goltz in 1960 as the association of multiple nevoid basal-cell epitheliomas, jaw cysts and bifid ribs [1]. Subsequent clinical series expanded the phenotype to include odontogenic keratocysts, palmoplantar pits, calcification of the falx cerebri, skeletal malformations, craniofacial abnormalities, ovarian or cardiac fibromas, medulloblastoma and multiple basal cell carcinomas (BCCs) [2, 3, 4, 5, 6, 7].

The estimated prevalence of GGS varies from approximately 1/57,000 to 1/256,000, with an approximately equal sex distribution [5]. Birth prevalence in the United Kingdom has been estimated at around 1/19,000 [8]. Although Gorlin syndrome occurs across diverse ethnic groups — including early documented series in African American families — the frequency of basal cell carcinomas shows a consistent racial disparity, with BCCs reported in up to 90% of White patients compared to approximately 38–40% of Black patients, likely reflecting a protective effect of increased skin pigmentation.

The syndrome is most commonly caused by heterozygous pathogenic variants in *PTCH1* and less commonly by variants in *SUFU*; *PTCH2* variants have been reported, but their contribution to BCNS remains uncertain [9, 10, 11]. *PTCH1* encodes Patched-1, a receptor that normally inhibits Smoothened (SMO) within the Sonic Hedgehog pathway. Loss of *PTCH1* function releases SMO inhibition, activates GLI-mediated transcription and promotes cellular proliferation. This mechanism underlies the development of BCCs and other syndrome-associated tumors [4,5,9].

Although classical GGS is frequently discussed in relation to intragenic variants or small insertions and deletions, copy-number alterations involving the 9q22.3 chromosomal region represent an important mechanism. A subset of reported cases represents a contiguous gene deletion — the so-called 9q22.3 microdeletion syndrome — in which the deleted interval includes *PTCH1* together with adjacent genes; such patients may demonstrate the tumor predisposition features of GGS together with developmental delay, intellectual disability, craniofacial dysmorphism, hearing impairment, overgrowth, craniosynostosis, hydrocephalus, Wilms tumor susceptibility and congenital anomalies [12, 13, 14, 15, 16, 17, 18, 19, 20, 21, 22]. Establishing that a given deletion is truly a contiguous gene deletion, however, requires methods capable of defining its size and boundaries, such as chromosomal microarray or array-CGH; gene-targeted MLPA, which tests only for loss of specific *PTCH1* exons/probes, cannot by itself confirm involvement of neighboring genes. This distinction creates important diagnostic nuance between isolated *PTCH1-*related GGS and a genomically defined 9q22.3 contiguous gene deletion syndrome.

Dermatologists may be the first clinicians to suspect the diagnosis, particularly when multiple BCCs develop in childhood or adolescence. Because sporadic BCC is extremely uncommon in this age group, the presence of multiple lesions should prompt syndromic assessment, careful examination for palmar pits and extracutaneous findings, histopathologic confirmation and genetic evaluation. We present a 15-year-old female with multiple histologically confirmed BCCs, palmar pits and an MLPA-confirmed deletion involving *PTCH1* at 9q22.3, accompanied by developmental and congenital abnormalities. The aim of this report is to highlight the dermatologic presentation of a *PTCH1*-region deletion with GGS phenotype, to discuss the extracutaneous findings observed, and to address the diagnostic, genetic and surveillance implications — while being explicit about what the testing method used can and cannot establish.

## CASE PRESENTATION

A 15-year-old female patient with developmental disability, including a specific speech articulation disorder, was evaluated at the University Clinic of Dermatology in Skopje, North Macedonia because of multiple facial skin lesions — erosions, crusted lesions, scars, milia and papulo-nodular lesions — together with palmar pitting noted on examination (Figure 1 and 2).

**Figure 1.**
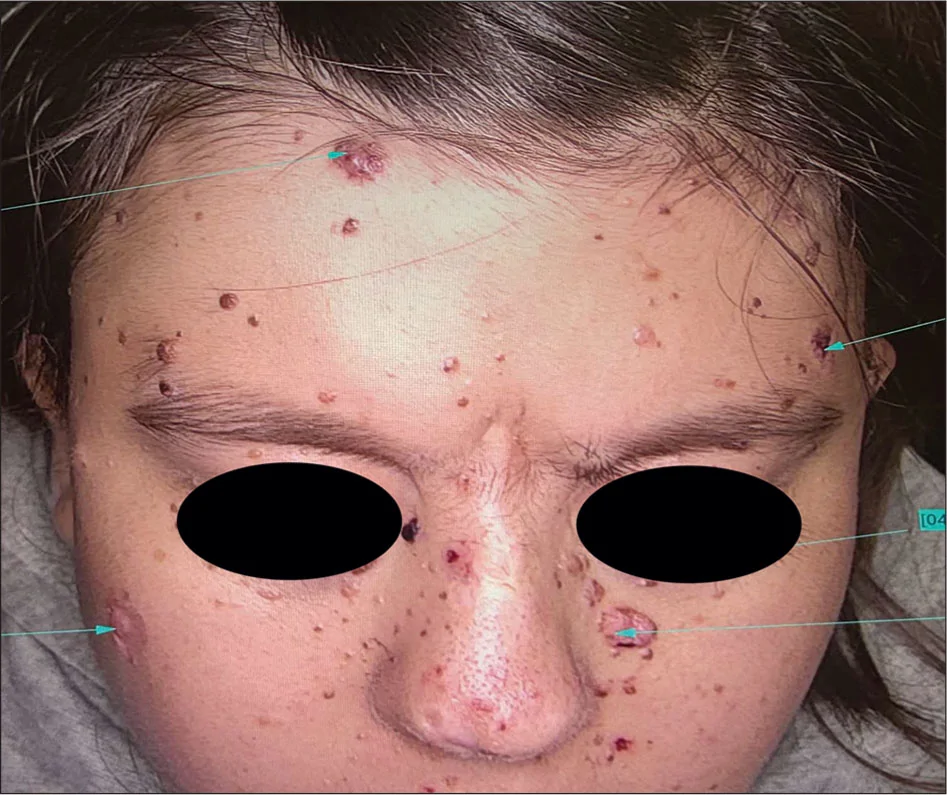
Clinical presentation showing multiple facial papules, nodules, erosions, crusts, scars and milia.

**Figure 2.**
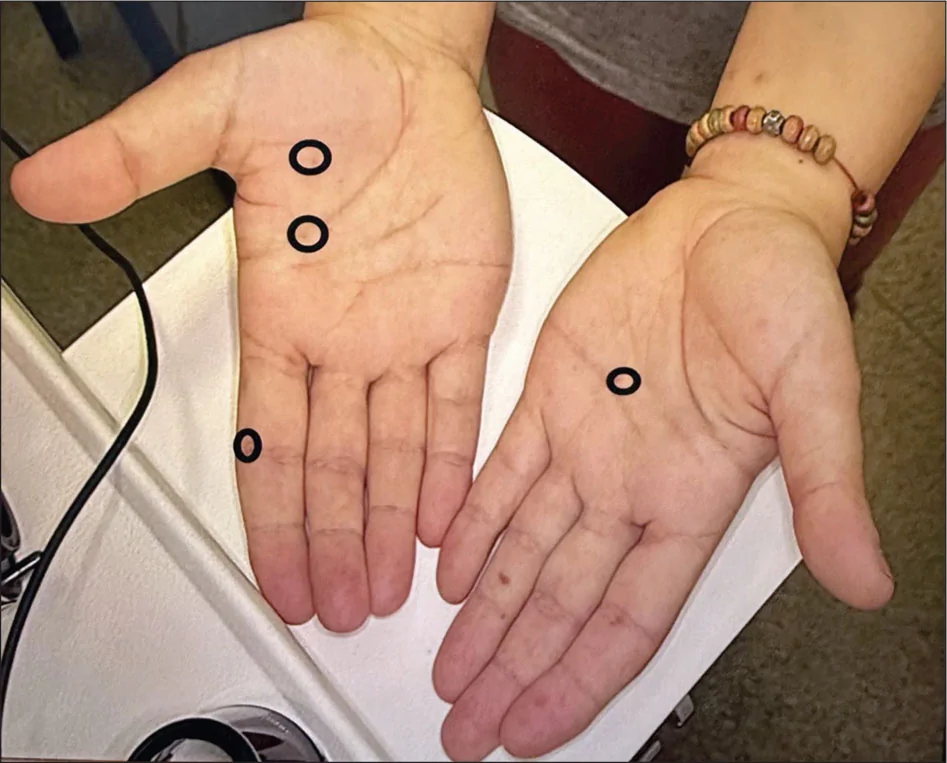
Palmar pitting observed on physical examination.

The occurrence of multiple clinically suspicious skin tumors in adolescence was considered unusual and raised suspicion for a hereditary BCC-predisposition syndrome. Dermoscopy of selected lesions revealed arborizing vessels, blue-gray ovoid nests, brown dots, ulceration, white scales and shiny white perpendicular lines. (Figure 3). These findings were considered consistent with BCC in the appropriate clinical context and guided selection of lesions for surgical management.

**Figure 3.**
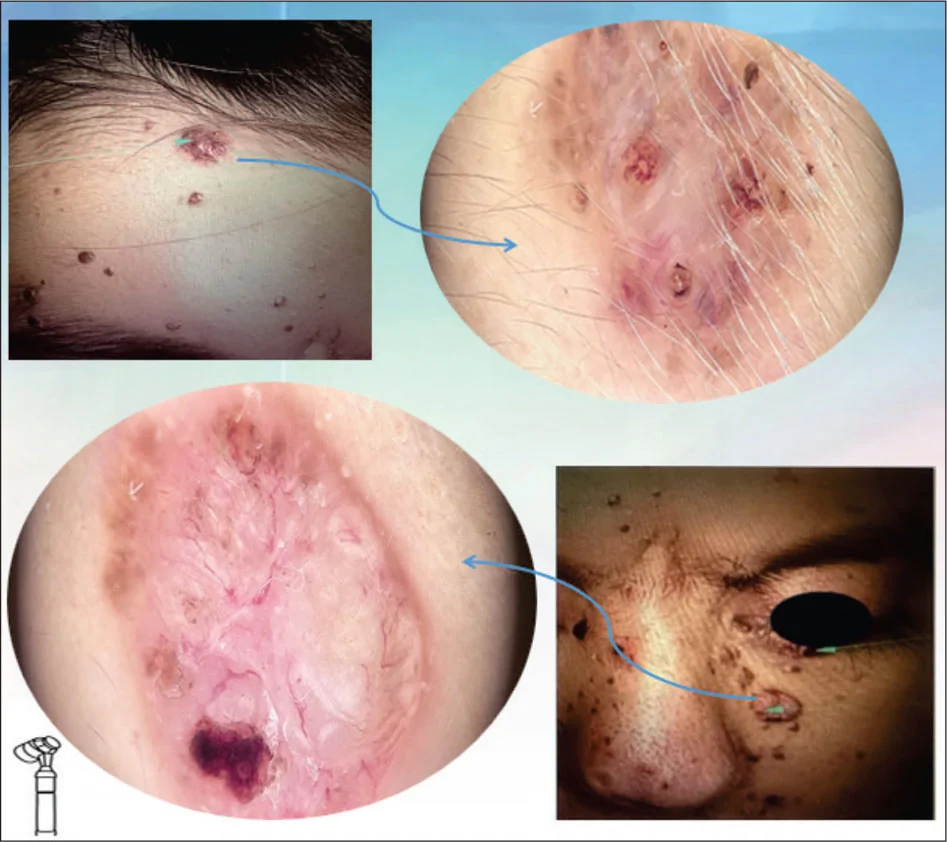
Dermoscopy of selected facial lesions demonstrating arborizing vessels, blue-gray ovoid nests, ulceration, white scales and shiny white perpendicular lines.

The patient was referred for surgical excision of selected lesions. Histopathological analysis confirmed basal cell carcinoma in three separate specimens. The final pathological diagnosis was carcinoma basocellular (Figure 4). Microscopic features reported in the clinical material included ulceration, atypical basaloid tumor cells infiltrating the dermis, peripheral palisading of tumor cells and associated lymphocytic inflammatory infiltrate. The histological confirmation of multiple BCCs at 15 years of age supported the suspicion of an inherited tumor predisposition syndrome.

**Figure 4.**
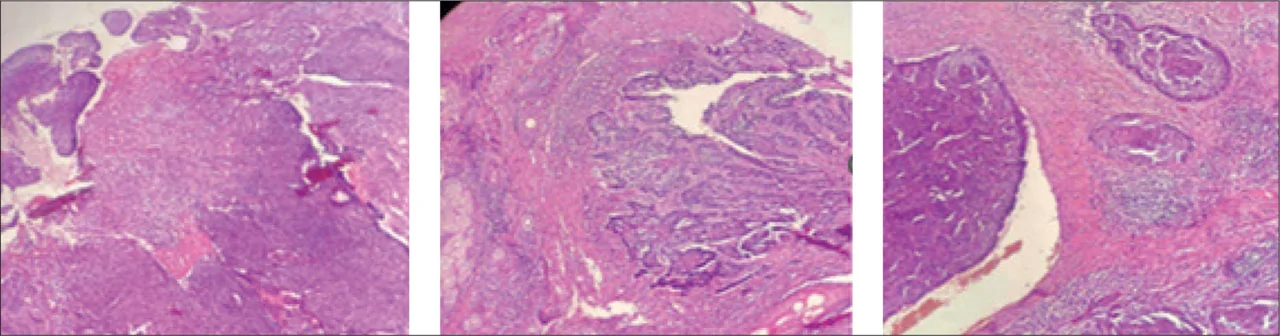
Histopathology of basal cell carcinoma showing dermal aggregates of atypical basaloid cells with peripheral palisading and inflammatory infiltrate. Hematoxylin and eosin stain, original magnification x200.

The patient’s past medical history revealed a complex phenotype. At approximately two years of age she had been surgically treated for a dermoid cyst located at the nasal root. Additional documented findings included strabismus, conductive hearing loss, kyphoscoliosis and a kinked aorta. The patient also had developmental disability with a specific speech articulation disorder, as mentioned before. Genetic testing was performed at the Research Centre for Genetic Engineering and Biotechnology “Georgi D. Efremov,” Macedonian Academy of Sciences and Arts, Skopje. Multiplex ligation-dependent probe amplification (MLPA) was performed using the SALSA MLPA Probemix P245-A2 Microdeletion Syndromes-1 (MRC-Holland, Amsterdam, the Netherlands), a screening panel for recurrent microdeletion syndromes. The analysis demonstrated a deletion involving the 9q22.3 region, which contains *PTCH1.*

Because *PTCH1* is located within the 9q22.3 region, this result provided a plausible molecular basis for the patient’s early-onset multiple BCCs and palmar pits. The additional systemic findings — developmental disability with speech articulation disorder, conductive hearing loss, ocular abnormality, kyphoscoliosis, dermoid cyst and cardiovascular abnormality — raise the possibility that the phenotype extends beyond classical isolated GGS. However, since the MLPA probemix used targets *PTCH1* specifically and does not interrogate flanking genomic regions, this dataset cannot confirm or exclude involvement of neighboring genes, and the broader “9q22.3 deletion syndrome” interpretation should be regarded as a differential consideration rather than a confirmed diagnosis pending array-based testing.

## DIAGNOSTIC ASSESSMENT

Contemporary diagnostic frameworks for BCNS/GGS evolved from criteria proposed by Evans and colleagues, modified by Kimonis and colleagues and revised by later consensus statements and guidelines [2,3,6,7]. Diagnosis may be established by one major criterion with molecular confirmation, two major criteria, or one major and two minor criteria [7]. Major criteria include multiple BCCs or BCC before young adulthood, odontogenic keratocysts, palmar or plantar pits, lamellar calcification of the falx cerebri, desmoplastic medulloblastoma and a first-degree relative with BCNS. Minor criteria include rib anomalies, macrocephaly, cleft lip or palate, ovarian or cardiac fibroma, lymphomesenteric cysts, ocular anomalies and specific skeletal malformations such as vertebral anomalies or kyphoscoliosis [7].

The present patient fulfilled major diagnostic elements through multiple BCCs before the age of 20 years and palmar pitting. Ocular abnormality and kyphoscoliosis represented supportive minor features ( Table 1). Molecular testing demonstrated a deletion involving the 9q22.3 chromosomal region, which contains *PTCH1*. However, because the available MLPA assay does not delineate the exact genomic boundaries of the deletion, the findings cannot establish whether *PTCH1* alone or additional neighboring genes are deleted. The clinical phenotype is therefore best described as a Gorlin-Goltz phenotype associated with an MLPA-detected *PTCH1*-region deletion at 9q22.3 (Table 2).

**Table 1.** Diagnostic criteria relevant to the present patient.

**Criterion type**	**Examples from guideline**	**Present patient**
Major criteria	Multiple BCCs before young adulthood; palmar or plantar pits; odontogenic keratocysts; falx cerebri calcification; desmoplastic medulloblastoma; first-degree relative with BCNS	Multiple BCCs at age 15; palmar pits
Minor criteria	Ocular abnormalities; skeletal anomalies including kyphoscoliosis; macrocephaly; rib anomalies; cardiac/ovarian fibroma; cleft lip/palate	Strabismus; kyphoscoliosis
Molecular support	*PTCH1* or SUFU pathogenic variant; copy-number loss may be relevant when phenotype is broader than classical GGS	MLPA-detected deletion involving 9q22.3, the region containing *PTCH1*

**Table 2.** Classical GGS, 9q22.3 deletion syndrome and the present patient.

**Feature**	**Classical GGS**	**9q22.3 deletion syndrome**	**Present patient**
Genetic mechanism	Usually *PTCH1, SUFU* or *PTCH2* sequence variant	Copy-number deletion involving 9q22.3, often including *PTCH1*	MLPA deletion involving 9q22.3
Multiple BCCs	Common	Reported, especially with PTCH1 loss	Present, histologically confirmed
Palmar pits	Common	Possible	Present
Odontogenic keratocysts	Common	Variable	No available data
Falx cerebri calcification	Common	Variable	No available data
Developmental disability	Not a dominant typical feature	Common	Present, with speech articulation disorder
Hearing loss	Uncommon	Reported	Conductive hearing loss
Cardiovascular findings	Cardiac fibroma may occur	Congenital anomalies may occur	Kinked aorta
Skeletal abnormalities	Rib/vertebral anomalies, kyphoscoliosis	Reported	Kyphoscoliosis
Dermoid cyst	Not classical	May occur in broader syndromic contexts	Nasal-root dermoid cyst

## DISCUSSION

The central diagnostic issue in this case is the distinction between classical GGS and 9q22.3 microdeletion syndrome. Both may involve *PTCH1* haploinsufficiency and both may produce early-onset or multiple BCCs, palmar pits and other GGS-associated findings. In many reported cases, molecular characterization has demonstrated deletions encompassing *PTCH1* and additional neighboring genes; however, the size and gene content of the deletion vary considerably among patients [12, 13, 14, 15, 16, 17, 18, 19, 20, 21, 22]. The phenotypic spectrum reported in association with 9q22.3 deletions is variable and appears to depend on deletion size — an inference drawn from array-based studies that our MLPA-only data cannot independently reproduce.

Early-onset multiple BCCs are a powerful clue to an inherited predisposition. GeneReviews describes NBCCS as being characterized by jaw keratocysts often beginning in the second decade and/or BCCs, along with facial milia and skeletal anomalies [9]. The 2022 clinical guideline for BCNS emphasizes that BCC before young adulthood or multiple BCCs is a major diagnostic criterion and recommends regular dermatologic surveillance, intensified after the first BCC [7]. In the present patient, multiple histologically confirmed BCCs at age 15 constitute a striking dermatologic manifestation.

The dermoscopic findings further support the diagnosis. Arborizing vessels, blue-gray ovoid nests, ulceration and shiny white structures are well-recognized dermoscopic features of BCC [23, 24, 25, 26]. Dermoscopy is particularly useful in syndromic contexts because patients may develop numerous lesions, including BCCs, milia, cysts, follicular hamartomas and inflammatory or traumatic erosions. It can help select lesions for biopsy or excision and can reduce unnecessary procedures during lifelong follow-up.

The published 9q22.3 microdeletion literature provides a useful comparative framework for interpreting this patient’s extracutaneous findings, even though our own molecular data cannot confirm a contiguous deletion. Muller and colleagues described a syndrome including metopic craniosynostosis, hydrocephalus, macrosomia and developmental delay in individuals with array-confirmed 9q22.3 microdeletion [12]. Shimojima and colleagues described clinical features of array-confirmed 9q22.3 microdeletion involving *PTCH1* [13], and Yamamoto and colleagues further delineated 9q22 deletion syndrome associated with basal cell nevus syndrome [14]. De Ravel and colleagues emphasized early non-GGS manifestations in patients with chromosome 9q22.32q31.1 microdeletions including *PTCH1* [15]. Musani and colleagues reported a Gorlin syndrome patient with a large deletion in 9q22.32–q22.33 [16]. Ewing and colleagues described a minimal deletion removing *PTCH1*, supporting the concept that deletion size influences phenotype [21]. All of these comparator studies relied on array-based or otherwise boundary-defining methods, in contrast to the gene-targeted MLPA used in our patient.

Oncologic implications of 9q22.3 deletions extend beyond BCC. Isidor and colleagues reported Wilms tumor in patients with array-confirmed 9q22.3 microdeletion and suggested a role for *PTCH1* in nephroblastoma [17]. Garavelli and colleagues described multiple tumor types, including leiomyoma and Wilms tumor, in a patient with 9q22.3 microdeletion encompassing *PTCH1* and FANCC [18]. Cayrol and colleagues subsequently reviewed Wilms tumor associated with the 9q22.3 microdeletion syndrome [19]. Although Wilms tumor was not documented in our patient, these reports support the need for genetic consultation, consideration of array-based confirmatory testing, and individualized surveillance based on deletion size, patient age and local expert recommendations.

The case most directly comparable from a dermatologic standpoint is the report by Kieny and colleagues, who described a patient with 9q21.33q22.3 microdeletion, a phenotype typical of 9q22.3 microdeletion syndrome and multiple BCCs treated with vismodegib [22]. This report shows that the dermatologic burden of BCC in 9q22.3 deletion syndrome may be clinically significant and may require advanced therapies. Our case adds an adolescent presentation with dermoscopic and histopathologic documentation. (Table 3)

**Table 3.** Selected 9q22.3/PTCH1 deletion literature relevant to the present case.

**Reference**	**Year**	**Main contribution**	**Relevance**
Muller et al. [12]	2012	Defined 9q22.3 microdeletion phenotype including craniosynostosis, hydrocephalus, macrosomia and developmental delay	Supports broader developmental phenotype(array-confirmed)
Shimojima et al. [13]	2009	Reported clinical features of array-confirmed microdeletion 9q22.3 involving *PTCH1*	Supports *PTCH1-*containing deletion phenotype
Yamamoto et al. [14]	2009	Further delineated 9q22 deletion with basal cell nevus/Gorlin association	Supports overlap concept
de Ravel et al. [15]	2009	Highlighted early non-Gorlin findings in *PTCH1*-containing microdeletion	Supports syndromic interpretation
Isidor et al. [17]	2013	Associated array-confirmed 9q22.3 microdeletion with Wilms tumor risk	Relevant to genetic counseling and surveillance
Ewing et al. [21]	2021	Minimal *PTCH1* deletion case	Supports genotype-phenotype correlation
Kieny et al. [22]	2018	Multiple BCCs in array-confirmed 9q22.3 microdeletion treated with vismodegib	Closest dermatologic comparator

The differential diagnosis of multiple BCCs in adolescence includes GGS, xeroderma pigmentosum, Bazex-Dupre-Christol syndrome, Rombo syndrome, multiple hereditary infundibulocystic BCC syndrome and generalized basaloid follicular hamartoma syndrome. Xeroderma pigmentosum is typically associated with marked photosensitivity and multiple ultraviolet-induced malignancies. Bazex-Dupre-Christol syndrome is characterized by follicular atrophoderma, hypotrichosis, milia and BCCs. Rombo syndrome may include vermiculate atrophoderma, cyanotic erythema, hypotrichosis and BCCs. In this patient, palmar pitting and an MLPA-confirmed *PTCH1*-region deletion strongly support a *PTCH1*-related disorder, while developmental disability and congenital findings raise — without confirming — the possibility of a broader deletion.

Management requires multidisciplinary coordination. Surgical excision remains standard for many BCCs. Topical treatments, photodynamic therapy or destructive modalities may be considered for selected low-risk superficial lesions, depending on local practice and histologic subtype. Hedgehog pathway inhibitors such as vismodegib and sonidegib are important options for locally advanced, metastatic or surgically intractable BCC, and they have been reported in patients with GGS or high-frequency BCC [27, 28, 29, 30, 31]. However, in pediatric or adolescent patients, the indication must be individualized by specialists because adverse effects and effects on development and growth require careful consideration. Radiotherapy should generally be avoided when possible in GGS because ionizing radiation can provoke numerous BCCs in irradiated fields [7,9].

For this patient, dermatologic follow-up every three to six months after the first BCC would be defensible, with dermoscopy used to monitor existing lesions and identify new tumors early [7]. Dental or maxillofacial assessment is appropriate to evaluate for odontogenic keratocysts, and review of cranial imaging is useful for falx cerebri calcification or other neurodevelopmental findings. Ophthalmologic and audiologic follow-up are indicated because of strabismus and hearing loss. Speech-language pathology follow-up is warranted given the documented articulation disorder. Cardiology follow-up is reasonable given the kinked aorta. Clinical genetics should guide family counseling and determine whether array-based testing (chromosomal microarray or array-CGH) would be useful to define the deletion boundaries.

## LIMITATIONS

The main limitation of this report is the absence of chromosomal microarray, array-CGH, or another genome-wide copy-number assay capable of defining the exact size and genomic boundaries of the deletion. MLPA using the P245-A2 Microdeletion Syndromes-1 probemix identified a deletion involving the 9q22.3 region; however, the assay does not permit precise delineation of the deletion boundaries or determination of all genes included within the deleted interval. Therefore, the contribution of *PTCH1* or other neighboring genes to the patient’s extracutaneous phenotype cannot be established from the available molecular data.

In addition, information regarding odontogenic keratocysts, calcification of the falx cerebri, long-term dermatologic follow-up, and family genetic testing was unavailable. Beyond the documented speech articulation disorder, detailed formal assessment of the patient’s broader developmental and neurobehavioral phenotype (including overall severity level and screening for autistic traits) was also limited. These limitations are acknowledged to avoid overinterpretation of the molecular and clinical findings.

## CONCLUSION

This case highlights a clinically important Gorlin-Goltz phenotype associated with an MLPA-detected deletion involving the 9q22.3 region. Multiple histologically confirmed BCCs at 15 years of age and palmar pitting strongly supported a diagnosis within the Gorlin syndrome spectrum, while developmental disability with speech articulation disorder, conductive hearing loss, strabismus, kyphoscoliosis, dermoid cyst, and cardiovascular abnormality suggested a broader syndromic phenotype.

The MLPA finding provides molecular evidence of a deletion involving the 9q22.3 region, which contains *PTCH1*. However, because the available assay does not define deletion boundaries or the complete gene content of the deleted interval, a specific contiguous-gene deletion and the individual contribution of neighboring genes cannot be established.

Dermatologists should consider inherited tumor-predisposition disorders when evaluating adolescents with multiple BCCs. When cutaneous findings are accompanied by developmental or congenital abnormalities, genetic testing capable of detecting copy-number alterations should be considered. Recognition of a possible 9q22.3 deletion-associated Gorlin phenotype has implications for dermatologic surveillance, multidisciplinary assessment, genetic counseling and long-term care.
